# A Machine Learning Approach for Walking Classification in Elderly People with Gait Disorders

**DOI:** 10.3390/s23020679

**Published:** 2023-01-06

**Authors:** Abdolrahman Peimankar, Trine Straarup Winther, Ali Ebrahimi, Uffe Kock Wiil

**Affiliations:** Centre of Health Informatics and Technology, The Mærsk Mc-Kinney Møller Institute, University of Southern Denmark, 5230 Odense, Denmark

**Keywords:** accelerometer, elderly population, gait disorders, healthcare monitoring, machine learning, medical AI

## Abstract

Walking ability of elderly individuals, who suffer from walking difficulties, is limited, which restricts their mobility independence. The physical health and well-being of the elderly population are affected by their level of physical activity. Therefore, monitoring daily activities can help improve the quality of life. This becomes especially a huge challenge for those, who suffer from dementia and Alzheimer’s disease. Thus, it is of great importance for personnel in care homes/rehabilitation centers to monitor their daily activities and progress. Unlike normal subjects, it is required to place the sensor on the back of this group of patients, which makes it even more challenging to detect walking from other activities. With the latest advancements in the field of health sensing and sensor technology, a huge amount of accelerometer data can be easily collected. In this study, a Machine Learning (ML) based algorithm was developed to analyze the accelerometer data collected from patients with walking difficulties, who live in one of the municipalities in Denmark. The ML algorithm is capable of accurately classifying the walking activity of these individuals with different walking abnormalities. Various statistical, temporal, and spectral features were extracted from the time series data collected using an accelerometer sensor placed on the back of the participants. The back sensor placement is desirable in patients with dementia and Alzheimer’s disease since they may remove visible sensors to them due to the nature of their diseases. Then, an evolutionary optimization algorithm called Particle Swarm Optimization (PSO) was used to select a subset of features to be used in the classification step. Four different ML classifiers such as k-Nearest Neighbors (kNN), Random Forest (RF), Stacking Classifier (Stack), and Extreme Gradient Boosting (XGB) were trained and compared on an accelerometry dataset consisting of 20 participants. These models were evaluated using the leave-one-group-out cross-validation (LOGO-CV) technique. The Stack model achieved the best performance with average sensitivity, positive predictive values (precision), F_1_-score, and accuracy of 86.85%, 93.25%, 88.81%, and 93.32%, respectively, to classify walking episodes. In general, the empirical results confirmed that the proposed models are capable of classifying the walking episodes despite the challenging sensor placement on the back of the patients, who suffer from walking disabilities.

## 1. Introduction

Physical activity plays a major role in mental and physical health and well-being. The correlation between physical activity and mental and physical health is stronger in the older population. Inadequate physical activity is linked with mobility disorders, loss of independence, and lower muscle strength [1]. Chodzko-Zajko et al. [2] showed that the development of age-related disabilities and other health diseases can be prevented and delayed by having an active lifestyle. It is reported by the World Health Organization (WHO) that the level of fitness and functional health is generally higher in the physically active elderly population [3].

One of the suitable methods for measuring physical activity is accelerometry, which does not constrain the subjects. Furthermore, accelerometry is considered a reliable and cost-effective method for monitoring ambulatory motion under free-living conditions [4,5]. However, in order to be able to evaluate physical activities using accelerometry, an accurate classification of different activity types is required [4].

The latest technological advancements in wearable devices have made it feasible to monitor daily activities. The new sensors are more cost-effective and last longer in terms of battery life. In recent years, various research has been conducted to classify daily activities using accelerometer data [6,7,8,9,10,11,12,13]. However, most of the developed models were tested on datasets collected from young population [7,9,10,14,15,16,17]. There are also other studies that focus on the data acquired from older individuals [12,18,19,20,21,22]. It has been shown in the literature that the models trained using the data from younger and healthy individuals do not generalize well when it is tested on the data acquired from the elderly population, who suffer from different diseases and gait disorders. This results in a lower performance on the data from older adults, which prevents using the developed models to classify activities of older individuals in free-living conditions [23]. In most of the previous studies, it has been also a common practice to use different sensor placements in order to achieve an accurate activity classification [24]. Although most of these studies have obtained interesting outcomes on physical activity classification using younger adults’ data and various sensors’ placements, there are still some questions that need to be further investigated. For this purpose, in a collaboration with a municipality in Denmark, we investigated the possibility of improving the quality of daily life in older citizens and patients, who suffer from dementia and walking difficulties. Analyzing the walking activity is of great importance for personnel in the municipality’s care home to monitor the physical health and for early detection of the development of dementia and/or other diseases in the individuals. However, due to the nature of dementia disease and its effect on the walking ability of the patients, it has been challenging to detect the walking activity of the individuals in the care home. Dementia usually affects the walking abilities of patients and forces them to use walking aids, which subsequently alters their walking patterns. Therefore, we try to investigate the following questions in this study:

- Is it applicable to develop a model that can classify the walking activity of patients with walking abnormalities, who suffer from dementia and Alzheimer’s disease?
- Is it possible to classify walking activities using only one sensor placed on the back of participants?
- How do the different Machine Learning (ML) algorithms perform on classifying walking (as one of the most effective and popular forms of activities) from non-walking activities in older adults?

To investigate the above points, in this paper, different ML algorithms are compared and presented, which are capable of classifying the walking activity of patients with walking disabilities using only one sensor placement on the back. It has been reported in the literature that gait disorders are correlated with Alzheimer’s disease [25,26]. The back sensor placement is desirable in patients with dementia and Alzheimer’s disease because they may remove the sensors placed on the other parts of the body, which are visible to them due to the nature of their diseases. In addition, using only one sensor placed on the back makes it possible to easily implement such a model in practice, especially in cases that are challenging to place multiple sensors such as on the thigh and hip. This is a huge advantage that removes the burdens of applying such models in practice in care homes/rehabilitation centers.

Two single and two ensemble classification algorithms were developed and evaluated using a dataset collected from older adults, who live in one of the municipalities in Denmark. The dataset contains 20 elderly patients, most of whom suffer from dementia and Alzheimer’s disease. To the best of our knowledge, there have not been any studies applying sensor placement on only the upper/mid back of patients with various walking difficulties as presented in this study. It has been proven that the performance of single classifiers can be enhanced using an ensemble learning framework. This is due to the fact that, in an ensemble approach, a collection of classifiers contributes to the final decision-making instead of only using a single weak learner. Thus, the performance of ensemble learning models is generally higher than single classification algorithms [27]. There are various applications in which ensemble learning methods are utilized such as cyber security [28,29,30,31,32,33], energy [34,35,36,37], and health informatics [38,39,40,41,42,43,44,45,46,47].

A vast range of features such as statistical, temporal, and spectral, were extracted from the collected accelerometer time series and the best subset of them were selected using Particle Swarm Optimization (PSO) algorithm [48]. The selected features are used as inputs for the ML models to classify walking from non-walking activities. The four used ML classifiers are k-Nearest Neighbors (kNN), Random Forest (RF), Extreme Gradient Boosting (XGB), and Stacking Ensemble (Stack).

The remainder of this paper consists of three sections. Section 2 describes the methodology of the used approaches in this study. In Section 3, the obtained results in this study are presented and discussed, and lastly, Section 4 concludes the paper.

## 2. Materials and Methods

### 2.1. Dataset

**Figure 1:**
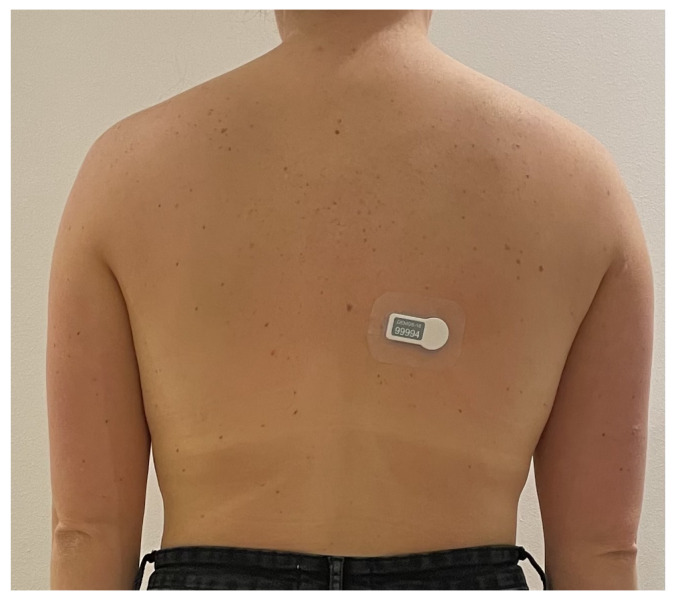
An example of sensor placement on the back of the subjects.

In this study, a dataset of 20 elderly patients, who live in one of Denmark’s municipalities and have different walking abnormalities, is used to train and evaluate the proposed model. It should be mentioned that The Regional Committee on Health Research Ethics for the Region of Southern Denmark was contacted regarding ethical approval of the study. They responded that according to Danish law about ethics related to health research, ethical committee approval was not required for this study. The dataset contains 20 time series, which are collected with a sampling frequency of 11 Hz, using accelerometer sensors developed by a Danish company called SENS Innovation ApS [49]. This is a commercial sensor that only measures the acceleration and temperature, which helps in having a longer battery lifetime. The sensor can record accelerometer data for up to two weeks without any recharging, which provides the opportunity for the healthcare professional to monitor the subjects longer. It is very easy to use and place the sensor on the back of the subjects. It is recommended by the manufacturer that the sensor should be placed on the mid back and slightly to the left or right of the spine as shown in Figure 1.

The length of the collected time series varies between around 278 s and 527 s per subject. The subject was asked to perform some free-living activities such as sitting and standing, sitting active, standing active, and finally walking around the care home for more than 10 m. The collected data is accompanied by a recorded video for each participant, which was used afterward for labeling the different activities performed by the participants. The participants in the study had dementia and Alzheimer’s disease history and they used different walking aids. There are 6 females and 14 males with an average age of 79.1 ± 6.9 and 76.4 ± 9.4, respectively. A summary of the dataset used in this study is given in Table 1. In general, the gender imbalance may lead to some classification biases. However, since the population size and the gender imbalance are not very large here, the investigation of gender imbalance is out of the scope of this paper. It should be noted that all the elderly patients in the municipality’s care home were asked to participate in this study without any specific criteria and the current population represents the ones who accepted the invitation.

### 2.2. Data Preprocessing

First, the triaxial accelerometer data (x-, y-, and z-axis) were segmented into smaller chunks of 3, 6, and 9 seconds with 50% overlaps between the adjacent segments. It should be mentioned that the size of segments was chosen due to the fact that the clinically proven length for human walking analysis is around 5-6 s. We evaluated half- and double-sized segment sizes (i.e., 3 and 9 s) as well to show that 6 s segment size is actually suitable for the purpose of our study. Then, each chunk (i.e., 3, 6, and 9 s) was labeled as either walking, if more than half of the samples are walking, or as non-walking (other activities) if the majority of the samples correspond to other activities.

**Figure 2:**
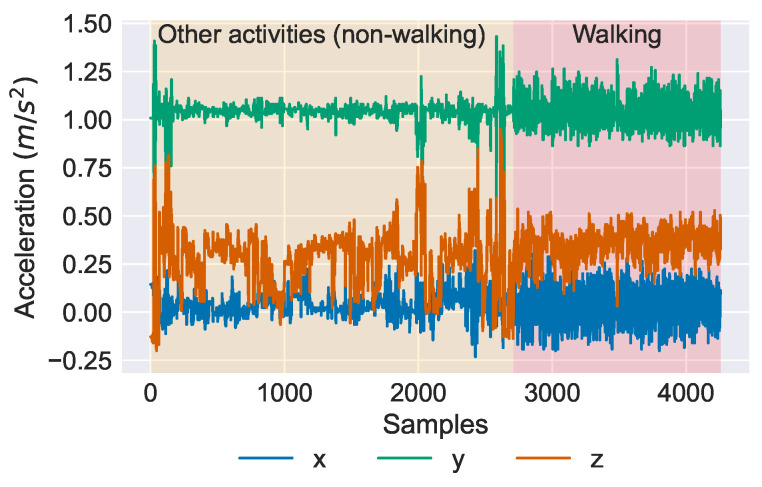
An example of collected accelerometer time series data for one of the subjects (#18). The other activities (non-walking) and walking parts for this specific subject are highlighted in yellow and red, respectively.

As an example, the triaxial accelerometer data for one of the subjects (# 18) is illustrated in Figure 2. As can be seen, the walking episode is relatively shorter compared to the non-walking (other activities) part. This results in a very imbalanced dataset. Therefore, a synthetic over-sampling method was also used to alleviate this problem and to increase the size of the walking class to be the same as the non-walking class. The oversampling process is explained in Section 2.7 in detail.

### 2.3. Feature Extraction

In this study, a Python package called Time Series Feature Extraction Library (TSFEL) [50] was used to extract various types of features. TSFEL is able to efficiently extract statistical, temporal, and spectral domain features. The computational complexities of the statistical, temporal, and most of the spectral features calculated by TSFEL are linear, which makes this library an efficient tool for the time series feature extraction task [50]. Table 2 provides a list of extracted features using TSFEL. The extracted features are briefly explained in Appendix A.

### 2.4. Feature Subset Selection

In order to improve the classification performance, a subset of defined features are usually selected to be used as inputs for the ML algorithms [51,52]. In this study, a PSO [48] was used to select the best and most accurate subset of features from all the features introduced in Section 2.3. The PSO is generally categorized as a swarm intelligence method. Swarm intelligence methods are based on the idea that single agents are not able to solve a problem individually. So, many agents (particles) try to achieve a unique goal in a swarm. In the PSO algorithm, each particle represents a potential solution for the problem at hand and every movement of the particles results in a new solution. The PSO algorithm works based on three rules: (1) the particles continue their movement in the same direction as the last movement (inertia term); (2) each particle should move towards its best-found solution (nostalgia term); and (3) each particle should also move towards the best solution, which has been found among all the particles (global term) [53,54]. The three rules used by the PSO algorithm for updating the position of the particles can be mathematically expressed as follows [55]:

(1) $$v_{i}\left(k\right)=\overset{\mathrm{inertia}\mathrm{term}}{\overset{︷}{\omega \cdot v_{i}\left(k-1\right)}}\;+\overset{\mathrm{nostalgia}\mathrm{term}}{\overset{︷}{c_{1}\cdot r_{1}\cdot \left(x_{{\mathrm{pbest}}_{i}}-x_{i}\left(k\right)\right)}}\;+\overset{\mathrm{global}\mathrm{term}}{\overset{︷}{c_{2}\cdot r_{2}\cdot \left(x_{{\mathrm{gbest}}_{i}}-x_{i}\left(k\right)\right)}}\;,$$

(2) $$x_{i}\left(k\right)=x_{i}\left(k-1\right)+v_{i}\left(k\right),$$

where ω is the inertia weight, $x_{{\mathrm{pbest}}_{i}}$ and $x_{{\mathrm{gbest}}_{i}}$ are the best-found position (solution) for particle $x_{i}$ and the global best solution of the swarm, respectively. The parameters $r_{1}$ and $r_{2}$ are random numbers in the range of [0, 1] and $c_{1}$ and $c_{2}$ are constants to control the nostalgia and global terms, respectively. In this paper, $c_{1}=c_{2}=2$ and ω=0.9 [56].

The overview of the PSO algorithm steps is described as follows:

- Initialize the positions and the velocities of the particles.
- Find and select the best particle ($x_{gbest}$) among the particles as leader.
- Repeat the following steps until the termination criteria is reached.
  – Update velocity (1).
  – Update position (2).
  – Find new $x_{pbest}$ for each particle.
  – Find new $x_{gbest}$ (leader).
  – Evaluate fitness function.
- Return the best particle as the most optimum solution.

### 2.5. Classification Models

For the classification part, four different algorithms were applied, which are introduced in the following subsections.

#### 2.5.1. k-Nearest Neighbors

kNN is considered a non-parametric classifier, which was first introduced by [57]. The kNN algorithm was further developed by Thomas Cover [58]. The output of the kNN algorithm is nothing but a class membership determined by the majority voting of its *k* neighbors. For example, if more than half of the neighbors vote for a class, the algorithm predicts that specific class as its final prediction. In addition, if k=1, then the sample is assigned the same class as that single nearest neighbor.

#### 2.5.2. Random Forest

RF is considered an ensemble learning method. For the training of RF, many different single decision trees (DTs) are built to make the final decision using a majority voting approach [59,60,61]. It should be mentioned that the single DTs in the RF classifier are created by random sub-sampling from the train data. In addition, a subset of variables/features is used to train each single DT. Using only a subset of available train data and features for building different DTs in the RF can also alleviate the over-fitting problem in the complex single DTs.

### 2.6. Extreme Gradient Boosting

Gradient boosted DTs is currently one of the mostly used algorithms in applied machine learning and XGB is a novel implementation of this algorithm, which is faster and has generally a higher performance compared to its predecessor [62]. A gradient boosting algorithm is used in XGB, which is categorized as an ensemble method [62]. Unlike the normal boosting algorithms in which the new trees are built to lower the errors of the previous ones, gradient boosting adds new trees/models to predict the errors of prior trees. Finally, all the individual trees are then combined to give the final prediction. It should be noted that the gradient descent algorithm is used in XGB to minimize the loss for adding new trees/models in the ensemble [63].

#### Stacking Ensemble

Stack is an ensemble learning method, which learns how to combine single classification algorithms to achieve the best overall performance. So, compared to other ensemble learning algorithms, such as RF and XGB, that use only DTs as the base learners, it can combine different types of classifiers. All the members of the ensemble generate a unique prediction for a specific sample. All the predictions from the single classifiers are then used as inputs for a meta estimator to make the final prediction [64,65,66]. Therefore, Stack is capable of combining different well-performing single classifiers in order to improve classification performance. In other words, it utilizes various single classifiers that are each good in different ways. Unlike RF and XGB, the whole train data is used to train the single learners and another model (meta estimator) is trained on top to learn the best possible combination of them [64]. However, it should be noted that the meta estimator, which is also named the level-1 model, is trained using the predictions made by the individual classifiers (level-0 models) on a subset of the dataset that has not been seen in the training step. In this paper, three single classifiers are used as level-0 models, which are kNN, RF, and XGB.

### 2.7. ML Based Walking Classification Framework

**Figure 3:**
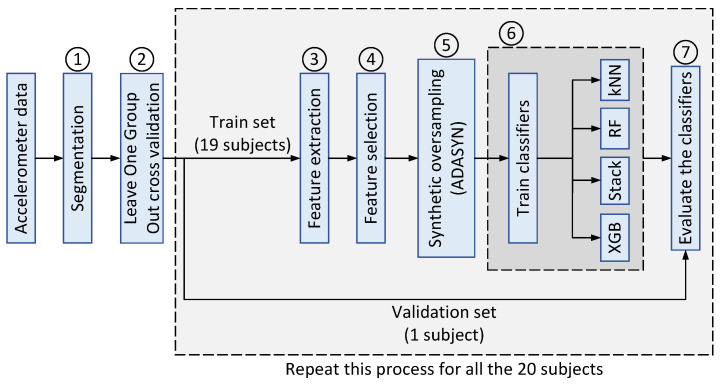
Flowchart of the proposed approach. The numbers correspond to the steps in Section 2.7.

**Figure 4:**
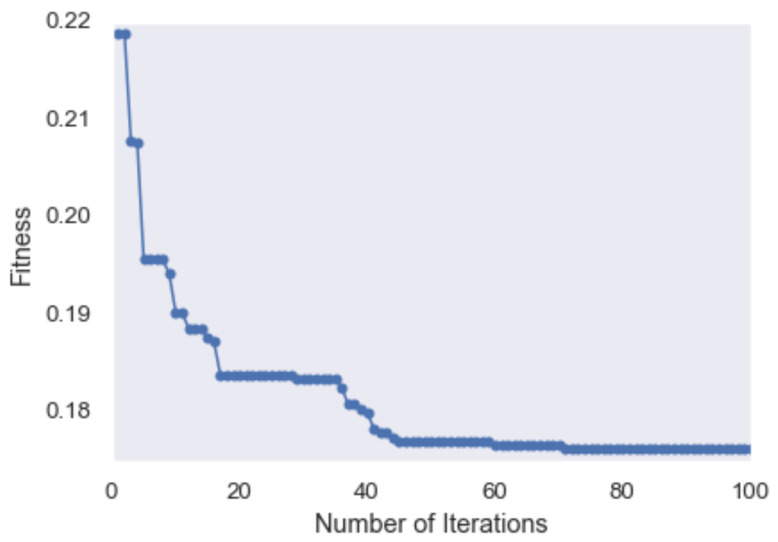
An example of PSO fitness curve.

In the proposed method, the capabilities of different ML classifiers are investigated and compared for walking classification using accelerometer data. The flowchart of the proposed approach is illustrated in Figure 3, which is described step by step as follows:

1. Segmentation: As mentioned in Section 2.2, the accelerometer time series are segmented into smaller chunks of 3, 6, and 9 s with 50% overlaps.
2. Cross validation: leave-one-group-out cross-validation (LOGO-CV) is applied to split the dataset into train and validation sets. In each iteration, all the individuals’ accelerometer data except one are used to train the classification algorithms. The remained subject/individual is then used to validate the models. This process is repeated twenty times in order to make sure that all the subjects are validated at least once.
3. Feature extraction: Three different types of features (i.e., statistical, temporal, and spectral), as listed in Table 2, are extracted from the segmented time series on all x-, y-, and z-axis. In total, 60 different features are extracted for each axis.
4. Feature selection: A subset of extracted features are selected using the PSO algorithm as explained in Section 2.4. The fitness (objective) function applied in the PSO algorithm is a combination of the error value and the size of the selected feature. The objective function is given in (3).
  (3) $$\mathrm{fitness}=\alpha \times \mathrm{error}+\beta \times \frac{\#\;\mathrm{of}\;\mathrm{selected}\;\mathrm{features}}{\#\;\mathrm{of}\;\mathrm{all}\;\mathrm{extracted}\;\mathrm{features}}.$$
  where α and β are the weight parameters equal to 0.9 and 0.1, respectively. Figure 4 shows the convergence of the PSO algorithm as the fitness value decreases by increasing the number of iterations.
5. Synthetic dataset oversampling: Since the length of walking episodes in the accelerometer data is shorter than the other activities (Figure 2), the number of extracted walking segments is smaller compared to non-walking segments. Therefore, the dataset is considered relatively imbalanced and the number of walking segments for training the classification algorithms is not sufficient. This increases the risk of a biased classification, which in turn leads to a higher error rate on the minority class (walking) [67]. To overcome this problem, the adaptive synthetic over-sampling technique (ADASYN) is applied to generate more samples for the minority class and to enable the classifiers to achieve their desired performance [67]. The ADASYN method consists of three main steps: (1) estimate the class imbalance degree to calculate the number of required synthetic samples for the minority class; (2) find the *K* nearest neighbors samples of the minority class using the well-known Euclidean distance; and (3) generate the synthetic samples for the minority class as follows:
  (4) $$d_{i}=x_{i}+\left(x_{ki}-x_{i}\right)\times \lambda ,$$
  where $x_{i}$ represents a sample from the minority class, $x_{ki}$ is one of the nearest neighbor samples chosen randomly, and λ∈[0,1] is a random value. As illustrated in Figure 3, the oversampling is only applied on the train set to avoid any unrealistic evaluation of the validation set.
6. Classifiers training: In this step, the preprocessed accelerometer data from nineteen subjects/individuals is used to train all the classification algorithms.
7. Classifiers evaluation: Finally, the performances of the four trained classifiers are evaluated using the validation set (Figure 3) to determine their performance.

## 3. Results and Discussion

**Figure 5:**
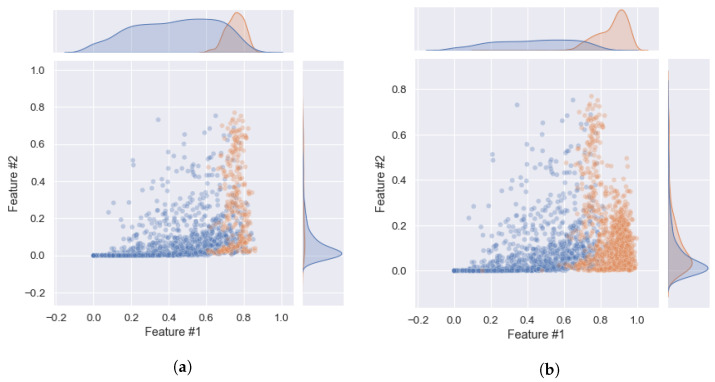
Scatter plots of two arbitrary features for walking and non-walking classes: (**a**) Imbalanced and (**b**) Balanced. The red and blue circles represent the Walking and Non-walking classes, respectively.

As stated in Section 2.1, a dataset of 20 elderly subjects is used to train and evaluate the performance of the different classifiers. In Section 2.7, we described the process of balancing the dataset using the ADASYN method. Figure 5a,b illustrate the distribution of the imbalanced and balanced data for two arbitrary features. It can be seen that the number of walking segments has been increased after applying ADASYN in Figure 5b. The number of segments for each class before and after data balancing are given in Table 3.

### 3.1. Evaluation Metrics

Various classification metrics can be utilized to compare and report the performance of the ML classifiers. A confusion matrix, as given in Table 4, can be used to calculate the different metrics. The rows and columns in Table 4 represent the actual labels and the predictions from the models, respectively. We use four classification metrics in this paper, which are accuracy (*Acc*), Sensitivity (*Se*), Precision or Positive Predictive Value (*PPV*), and *F*-score. The definitions of these metrics are given as follows using Table 4:

(5) $$Acc=\frac{TP+TN}{TP+FN+FP+TN},$$

(6) $$Se=\frac{TP}{TP+FN},$$

(7) $$PPV=\frac{FP}{FP+TN},$$

(8) $$F-score=\left(1+\beta \right)\frac{PPV\cdot Se}{(\beta^{2}\cdot PPV)+Se}.$$

where *TP*, *TN*, *FP*, and *FN* are the number of true positives, true negatives, false positives, and false negatives, respectively. *F*-score is actually a weighted harmonic mean of *Se* and *PPV*. If β=1, it is called a balanced *F*-score (*F*_1_-score), which takes into account both *Se* and *PPV* equally.

### 3.2. LOGO-CV Classification Performance

**Figure 6:**
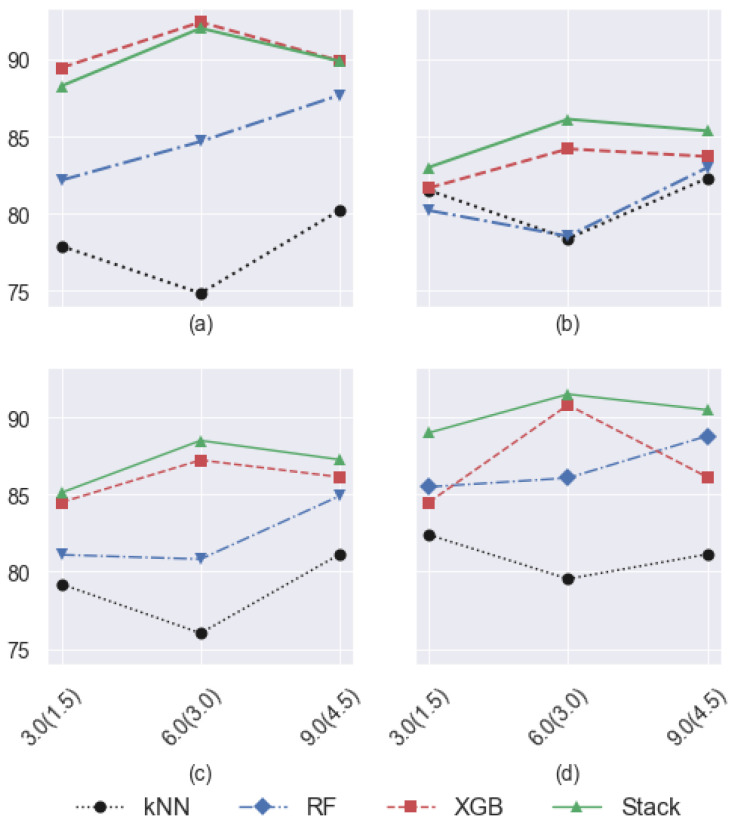
Comparison of the classifiers’ performance for the three different segment lengths: (**a**) PPV, (**b**) Se, (**c**) F_1_-score, and (**d**) Acc. The x-axis represents the segment’s length with their corresponding overlaps in the parentheses, i.e., S(O).

The average performance of the different classification algorithms on 20 subjects for *S* = 3, 6, and 9 s are plotted in Figure 6. As can be seen, the kNN model has the lowest performance among all the classifiers for almost all the segment lengths. On the other hand, the Stack model achieves the highest ACC, Se, and F_1_-score. In terms of PPV (precision), the XGB classifier outperforms other models for *S* = 3 s (Figure 6a). However, the precision of the Stack model is comparable with the XGB for *S* = 6 and *S* = 9 seconds. From Figure 6, it can be concluded that *S* = 6 s is the most optimum segment length for the detection of walking episodes. Therefore, we present some of the obtained results for *S* = 6 s in the rest of this section. The window size of 5–6 s is also considered clinically valid for human walking analysis [12].

Table 5 summarizes the average performance over all the subjects for the four classifiers (*S* = 6 s). These results confirm that the Stack method outperforms other classifiers on Se, F_1_-score, and Acc with 86.85%, 88.81%, and 93.32%, respectively. In addition, the PPV (precision) of XGB and Stack are comparable with 94.02% and 93.25%, respectively.

Table 6 reports the performance of the Stack classifier as the best method on all subjects. The selected features using the PSO algorithm are also given in the last column. The corresponding feature names can be found in Table 2. The total number of selected features for different subjects varies between 23 and 34 out of 60 extracted features. As given in Table 6, almost all the subjects achieved F_1_-score, PPV, and Acc above 90% except subject numbers 2, 5, 9, 10, and 14. However, subjects 9, 10, and 14 are the most challenging ones for the detection of walking episodes with Se of 75%, 73%, and 78% and PPV equal to 81%, 80%, and 74%, respectively. From Table 1, all these three subjects use a walker and they suffer from diseases such as spinal stenosis, hip fracture, osteoporosis, osteoarthritis, and knee problem, which makes it more difficult for them to walk normally.

### 3.3. Inter-Subjects Analysis

In this section, we evaluate the performance of the developed classifiers on five carefully chosen subjects with different walking aids. So, the classifiers were first trained on 15 subjects and they were then tested on the 5 remaining individuals. From Table 1, subjects number 3, 9, 13, 15, and 20 were selected as test sets. Subject 3 is the only one who uses a crutch, while numbers 9 and 13 use a walker and numbers 15 and 20 have no walking aids. This selection ensures that there are representatives from almost all groups in the test set, which makes the model evaluation more robust and reduces the classification bias on the unseen data in the future. In addition, the selected subjects are balanced in terms of gender with three males and two females.

The performance of the four classifiers for *S* = 3, 6, and 9 s is given in Table 7. In general, the Stack method performs the best for almost all the segment lengths, while XGB achieves slightly higher PPV compared to others. It is interesting to note that Stack and XGB outperform the other two classifiers, namely kNN and RF. As it can be seen from Table 7, the classification performance is higher for *S* = 6 s compared with other segment lengths. Stack is the most sensitive and accurate classifier with Se and Acc equal to 86.13% and 91.50% for *S* = 6 s, respectively. In addition, Stack achieves 88.50% for F_1_-score measure as the highest among all classification algorithms. The PPV value for the XGB method is the maximum at 92.42%, which is slightly higher than Stack with a PPV value of 92.03%.

**Figure 7:**
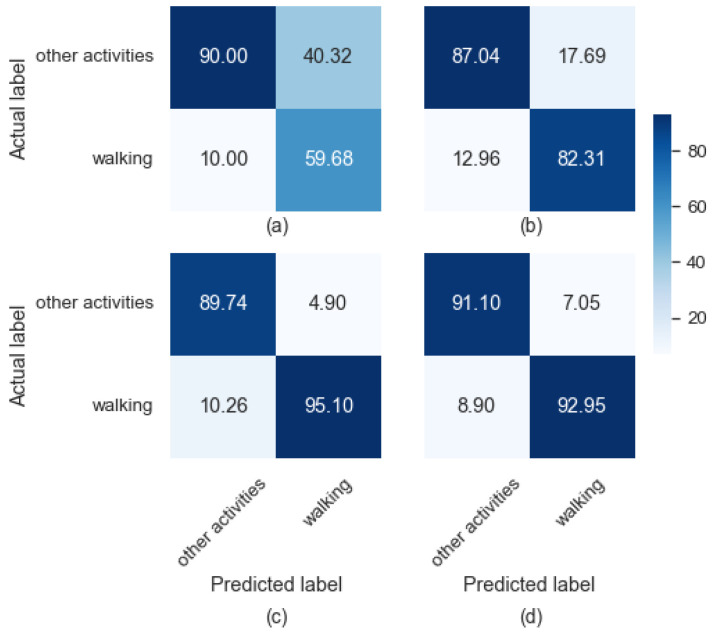
Confusion matrix of: (**a**) kNN, (**b**) RF, (**c**) XGB, and (**d**) Stack for *S* = 5 s. The numbers are in percentage. The color bar shows the actual number of segments that fall into different cells of the matrix.

The confusion matrices for the four classifiers on the five test subjects (3, 9, 13, 15, and 20) are given in Figure 7. The reported results are for *S* = 6 s and *O* = 3 s, which was shown to be the most optimum segment length as per the results in Table 7. For example, the Stack method detects most of the walking and non-walking segments correctly (Figure 7d), which are equal to 91.1% and 92.95% of the cases, respectively. In other words, only 8.9% of the walking segments are incorrectly classified into other activities (non-walking) compare to 10.26% for the XGB method. This makes Stack classifier a very suitable method for the detection of walking episodes from the accelerometer data. On the other hand, the number of false positive cases for the XGB method (4.9%) is the least among all the classifiers, which explains the higher PPV for XGB as reported in Table 7.

**Figure 8:**
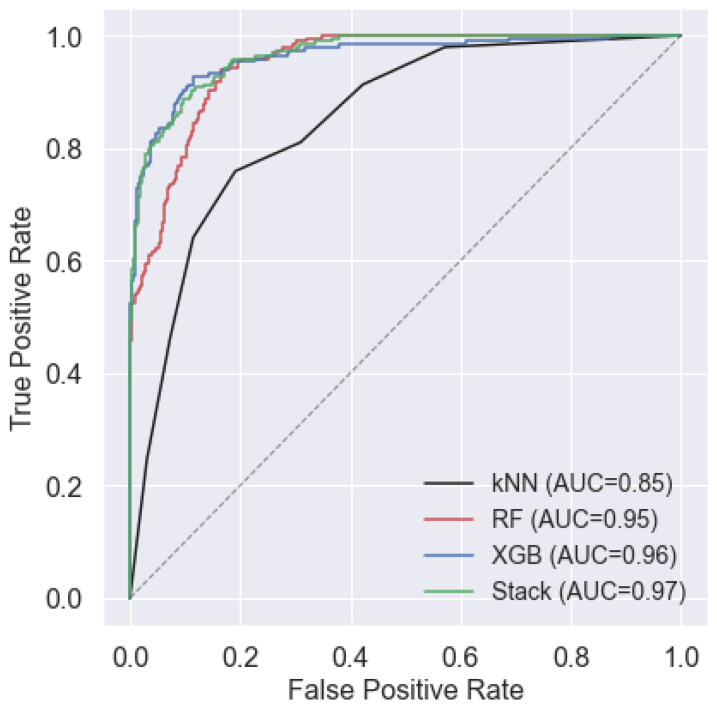
ROC curves for the proposed four different classification algorithms. The diagonal dashed line represents the random classification (chance).

The ROC curve of the proposed models for the five test subjects (3, 9, 13, 15, and 20) are also plotted in Figure 8. As shown in the figure, the Stack model achieves the highest area under the curve (AUC) of 0.97 followed by XGB and RF with 0.96 and 0.95, respectively. As expected, there is a considerable gap between kNN (AUC = 0.85) and other algorithms. This shows the promising classification performance for ensemble learning methods especially the Stack classifier as a combination of two ensemble-based methods (RF and XGB) and a single classifier (kNN).

### 3.4. Computational Resources

All the experiments presented in this paper were run on the University of Southern Denmark’s internal cloud infrastructure with 64 vCPU and 376 GB RAM. Overall, the processing time of one iteration of the LOGO-CV algorithm (i.e., training on 19 subjects and validation on 1 remaining patient) takes around 178.37 s. Several open-source libraries were used to conduct the experiments such as scikit-learn [68], XGBoost [62], NumPy [69], Pandas [70], Matplotlib [71]. It should be also noted that the size of the collected triaxial accelerometer data for 20 patients is around 10.5 MB.

## 4. Conclusions, Limitations, and Future Works

In this paper, different ML models have been developed to classify walking episodes from other activity types (non-walking). For this purpose, 60 different features (statistical, temporal, and spectral) were first extracted from the accelerometer data. Then, a PSO algorithm was applied to select the best subset of features, which were then used as inputs for the ML classifiers. There were three main contributions to this work which addressed the three questions stated in Section 1. First, the performances of different ML methods were compared for classifying walking segments in older adults. Second, we investigated the possibility of using the sensor placement only on the back, unlike the conventional hip and thigh or multi-sensor placement. Although placing the sensor on the back makes it more challenging to detect walking activity, it is most desired in cases where the subjects suffer from dementia and Alzheimer’s disease. Third, the proposed models were evaluated to whether they are suitable for classifying the walking activity of older subjects with walking abnormalities. The experimental results showed that the single classifiers such as kNN were outperformed by ensemble-based models (RF and XGB). In addition, the obtained results showed that the Stack model, which is a combination of all three classifiers (kNN, RF, and XGB), outperforms others. For example, Stack achieved improvement on Se, Acc, and F_1_-score by around 1%. From this, we can also conclude that the classification results improve by increasing the diversity of the classifiers included in the ensemble. For example, even though the kNN classifier is inferior to the ensemble classifiers, it helps the ensemble model (Stack) for the detection of walking episodes that are challenging for other methods such as RF and XGB. Therefore, the combined Stack model achieves the highest performance. The obtained results confirm that ML methods can be efficiently applied in clinical settings to classify walking segments using accelerometer data collected from elderly dementia patients, which paves the way to be used *in house* by personnel in care homes/rehabilitation centers to better monitor patients’ daily activities and progress.

There are also some limitations to our proposed algorithm, which can be studied in future works. First, the model’s performance highly depends on the feature extraction and a selection step. The performance of the model might be decreased in case of less ideal feature extraction and selection. So, the impact of the different feature extraction and selection techniques can be further investigated. Second, the population size is limited in this study. We may collect data from more municipalities’ care homes in the future. This enables us to train more advanced Deep Learning (DL) models that can handle raw accelerometer data, which subsequently helps to bypass the feature engineering step. Finally, the algorithmic bias was not investigated to study the effectiveness of the proposed model on a highly imbalanced population in terms of gender and age.

## Figures and Tables

**Table 1:** An overview of the dataset used in this study.

ID	Sex	Age	Walking Aids	Dementia Diagnosis
1	Male	90	Stick	Alzheimer’s disease
2	Male	82	None	Lewy body dementia
3	Female	85	Crutch	Unknown
4	Female	75	None	Alzheimer’s disease
5	Male	63	None	Lewy body dementia
6	Male	68	None	Alzheimer’s disease
7	Male	62	None	Alzheimer’s disease
8	Male	80	None	Dementia
9	Male	89	Walker	Unknown
10	Female	84	Walker	Unknown
11	Male	66	None	Unknown
12	Male	73	None	Parkinson’s disease
13	Male	79	Walker	Parkinson’s disease
14	Female	72	Walker	Unknown
15	Female	87	None	Alzheimer’s disease
16	Female	72	Walker	Vascular dementia
17	Male	90	None	Alzheimer’s disease
18	Male	79	None	Alcohol-related dementia
19	Male	79	None	Alzheimer’s disease
20	Male	68	None	Dementia

**Table 2:** A summary list of the extracted features used in this study.

Feature Domain		
**Statistical**	**Temporal**	**Spectral**
(1) Empirical cumulative distribution function	(17) Absolute energy	(35) Mean value of each spectrogram frequency
(2) ECDF percentile	(18) Total energy	(36) Fundamental frequency
(3) ECDF percentile count	(19) Centroid	(37) Human range energy ratio
(4) Histogram	(20) Area under the curve	(38) Linear prediction cepstral coefficients
(5) Interquartile range	(21) Autocorrelation	(39) Mel-frequency cepstral coefficients
(6) Minimum	(22) Shannon entropy	(40) Spectral positive turning points
(7) Maximum	(23) Mean absolute differences	(41) Spectral roll-off
(8) Mean	(24) Median absolute differences	(42) Spectral entropy
(9) Median	(25) Mean of differences	(43) Spectral roll-on
(10) Mean absolute deviation	(26) Median of differences	(44) Maximum power spectrum density
(11) Median absolute deviation	(27) Sum of absolute differences	(45) Maximum frequency
(12) Root Mean Square	(28) Positive turning points	(46) Median frequency
(13) Variance	(29) Negative turning points	(47) Power spectrum density bandwidth
(14) Standard Deviation	(30) Zero-crossing rate	(48) Spectral centroid
(15) Kurtosis	(31) Peak to peak distance	(49) Spectral decrease
(16) Skewness	(32) Traveled distance	(50) Spectral distance
	(33) Slope of signal	(51) Spectral kurtosis
	(34) Number of peaks from a subsequence	(52) Spectral skewness
		(53) Spectral slope
		(54) Spectral spread
		(55) Spectral variation
		(56) Wavelet absolute mean
		(57) Wavelet energy
		(58) Wavelet standard deviation
		(59) Wavelet entropy
		(60) Wavelet variance

**Table 3:** Number of segments for imbalanced and balanced datasets.

Class	Imbalanced	Balanced
Walking	20,456	70,779
Non-walking (other activities)	72,746	72,746

**Table 4:** Confusion matrix.

	Predicted Negative	Predicted Positive
Actual negative	True negative (TN)	False positive (FP)
Actual positive	False negative (FN)	True positive (TP)

**Table 5:** Average classification performance of the classifiers for all subjects for *S* = 6 s with *O* = 3 s. The numbers are in percentage and the highest performances of the different models are given in bold.

Algorithm	Se	F_1_-Score	PPV	Acc
kNN	77.38	76.58	74.95	79.75
RF	77.96	81.25	84.85	87.73
XGB	84.53	87.25	**94.02**	92.47
Stack	**86.85**	**88.81**	93.25	**93.32**

**Table 6:** Classification performance of the Stack method on the individual subjects. The selected features using the PSO algorithm are also reported in the last column. These results correspond to the *S* = 6 s with *O* = 3 s.

ID	Se	F_1_-Score	PPV	Acc	Selected Features Numbers (Table 2)
1	90	94	99	97	1–4, 8, 12, 13, 17, 19–21, 23, 35, 37–44, 52, 55–60
2	85	87	89	93	1–4, 6, 9–11, 14, 16, 17, 29, 31, 34, 35, 38, 39, 42–44, 48, 50–57, 60
3	90	93	97	97	1, 4, 10, 13, 21, 25, 28, 33–35, 37–42, 45, 48, 50, 52, 56–58, 60
4	86	92	98	98	1, 2, 4, 7, 9, 10, 15, 19, 20, 21, 25, 28, 29, 31, 35–38, 46, 47, 49, 52–58, 60
5	85	87	90	93	1, 3, 4, 6, 7, 9, 10, 13, 15-18, 21, 23, 31, 33, 35, 36, 38, 39, 41–43, 50, 51, 55–59, 60
6	90	91	92	94	1–6, 8, 12, 14, 23, 28, 29, 31, 33, 35-37, 39, 41, 44, 45, 47, 48, 50–53, 55-58, 60
7	93	96	100	98	1–4, 10, 12, 13, 16, 17, 18, 20, 21, 24, 30, 35–40, 43, 45, 47, 48, 50–52, 54–60
8	90	93	96	92	1, 3-5, 11, 13, 15, 16, 19, 29–31, 34–36, 38, 39, 43, 44, 48, 50–52, 54, 56–58, 60
9	75	72	81	73	1, 2, 4–7, 10, 21–23, 28, 31, 35, 36, 38, 39, 40, 41, 43, 48–51, 56–57, 60
10	73	75	80	81	1–5, 7, 9, 10, 13, 20–22, 23, 27, 28, 34–36, 38, 39, 53–56, 58–60
11	88	93	98	97	1, 2, 4, 16, 18–20, 22, 29, 30, 33–35, 38, 39, 41, 46, 50, 51, 54, 56–58, 60
12	90	94	98	98	1–4, 6, 9, 11, 13, 14, 15, 17, 20–24, 28, 33, 35–39, 41–43, 45, 49, 52, 56–58, 60
13	90	93	96	96	1–5, 8–11, 13, 15–17, 19–21, 26, 29, 34, 36, 38, 39, 49, 51–54, 56–58, 60
14	78	76	74	89	1, 3, 4, 6, 7, 9, 11, 14, 17, 18, 20, 22, 26–29, 31, 33, 38, 39, 41, 48, 49, 51, 56–58, 60
15	89	91	94	95	1, 4, 8, 10, 11, 13–15, 17–20, 25–27, 33–35, 38–41, 48, 49, 54, 57, 58, 60
16	90	92	95	95	1–4, 9, 10, 12, 14, 20, 23, 27, 28, 30, 31, 33, 37–39, 42, 47, 53, 56–58, 60
17	90	94	98	98	1–5, 8, 11–14, 17, 21, 30, 38, 39, 40, 41, 43, 45, 52, 54, 56, 57, 60
18	89	92	95	97	1, 3–6, 10, 12, 13–18, 21, 26, 28, 30, 34–39, 42, 45, 46, 51–54, 56–58, 60
19	90	94	98	98	1, 4, 5, 8–10, 12, 16, 18, 21, 24, 25, 35, 38, 39, 42, 43, 49, 50–53, 56–58, 60
20	89	93	97	94	1, 2, 4, 6, 7, 12, 15, 21, 22, 24–26, 28, 30, 31, 34, 35, 38, 39, 41, 43, 44, 47–50, 56–58, 60

**Table 7:** Performance comparison of different classifiers for *S* = 3, 6, and 9. The numbers are in percentage and the highest performances of the different models are given in bold.

Algorithm	*S* = 3				*S* = 6				*S* = 9			
	Se	F_1_-Score	PPV	Acc	Se	F_1_-Score	PPV	Acc	Se	F_1_-Score	PPV	Acc
kNN	77.89	79.19	77.89	82.39	78.39	76.01	74.84	79.53	82.30	81.12	80.22	84.57
RF	80.21	81.09	82.17	85.49	78.54	80.81	84.68	86.07	82.98	84.91	87.69	88.79
XGB	81.66	84.49	89.46	88.87	84.20	87.23	**92.42**	90.81	83.71	86.13	89.91	89.85
Stack	83.01	85.12	88.28	89.00	**86.13**	**88.50**	92.03	**91.50**	85.37	87.26	89.88	90.49

## Data Availability

According to Danish data protection laws, the collected data set cannot be shared outside the project.

## Appendix A. Extracted Features

### Appendix A.1. Empirical Cumulative Distribution Function

The empirical cumulative distribution function (*ECDF*) is calculated by first ordering all the available unique values in the time series and then the cumulative probability is computed for each value/observation as follows:

(A1) $$ECDF\left(x\right)=\frac{\left(\#\;\mathrm{of}\;\mathrm{observations}\leq x\right)}{n},$$

where the nominator represents the number of observations/values that are less than or equal to a given value such as *x* and *n* is the total number of values in the time series data.

### Appendix A.2. ECDF Percentile and ECDF Percentile Count

ECDF percentile calculates the 0.2 and 0.8 percentiles of the ECDF as below:

(A2) $$ECDF_{percentile}=\frac{\#\;\mathrm{of}\;\mathrm{values}\;\mathrm{below}\;\mathrm{the}\;\mathrm{desired}\;\mathrm{percentile}}{n},$$

where the nominator computes the number of the observations/values that are less than the desired percentile and *n* is the total number of values in the time series data. Similarly, ECDF percentile count computes the cumulative sum of samples that are less than the percentile

### Appendix A.3. Histogram

Histogram represents the probability of occurring certain values. It is done by defining a number of bins with certain length to calculate the probability of the values occurrences in that specific bin.

### Appendix A.4. Interquartile Range

The interquartile range (IQR) simply calculates the difference between the 0.75 and 0.25 percentile of the data.

### Appendix A.5. Minimum, Maximum, Mean, and Median Value

The minimum and maximum are simply the greatest and smallest value of the time series (signal). However, the mean and median values of a signal can be calculated as below:

(A3) $$Mean\left(X\right)=\frac{x_{1}+x_{2}+\cdots +x_{n}}{n},$$

and

(A4) $$Median\left(X\right)=\left\{\begin{array}{cc} x_{\left(\frac{n+1}{2}\right)}, & \mathrm{if}\;n\;\mathrm{is}\;\mathrm{odd}\\ \frac{x_{\left(\frac{n}{2}\right)}+x_{(\frac{n}{2})+1}}{2}, & \mathrm{if}\;n\;\mathrm{is}\;\mathrm{even} \end{array}\right.$$

where *X* represents the signal with its size equal to *n*, i.e., $X=\{x_{1},x_{2},\cdots ,x_{n}\}$.

### Appendix A.6. Mean Absolute Deviation and Median Absolute Deviation

Mean absolute deviation of a time series is defined as the average distance of each point from the mean of the time series. This shows the degree of variability in the time series. Median absolute deviation computes the spreadness degree of the data points in a time series. The mean and median absolute deviation can be calculated in (A5) and (A6).

(A5) $$\mathrm{Mean}\;\mathrm{absolute}\;\mathrm{deviation}\;=\frac{1}{n}\sum_{i=1}^{n}\left|x_{i}-Mean\left(X\right)\right|,$$

(A6) $$\mathrm{Mean}\;\mathrm{absolute}\;\mathrm{deviation}\;=Median(|x_{i}-Median\left(X\right)|),\;i=1,2,\cdots ,n,$$

where Mean and Median are the mean and median of the time series defined in (A3) and (A4).

### Appendix A.7. Root Mean Square

Root mean square of a signal (time series) is the square root of the mean squares of the data points, which can be calculated as:

(A7) $$\mathrm{Root}\;\mathrm{means}\;\mathrm{quare}\;=\sqrt{\frac{1}{n}\left(x_{1}^{2}+x_{2}^{2}+\cdots +x_{n}^{2}\right)},$$

where *n* is the size of the time series.

### Appendix A.8. Variance and Standard deviation

Variance measures the variability of the data points from the mean and standard deviation is the square root of the variance. The variance and standard deviation are computed as follows:

(A8) $$Variance=\frac{1}{n-1}\sum_{i=1}^{n}\left(x_{i}-Mean{\left(X\right)}^{2}\right),$$

(A9) $$\mathrm{Standard}\;\mathrm{deviation}\;=\sqrt{Variance},$$

where *n* and Mean are the size and average of the time series *X*.

### Appendix A.9. Kurtosis & Skewness

Both Kurtosis and Skewness describe the shape of a distribution, which are computed as:

(A10) $$Skewness=\sum_{i=1}^{n}{\left(\frac{x_{i}-Mean\left(X\right)}{Variance}\right)}^{3},$$

and

(A11) $$Kurtosis=\sum_{i=1}^{n}{\left(\frac{x_{i}-Mean\left(X\right)}{Variance}\right)}^{4},$$

where Mean and Variance are given by (A3) and (A8).

### Appendix A.10. Absolute and Total Energy, Centroid, and Area under the Curve

Absolute energy is the sum of the squares of data points, which is computed as given in (A12). However, total energy is the absolute energy divided by time range of the time series as given in (A13). Similarly, centroid of a time series along the time axis is calculated in (A14). Area under the curve of a time series is simply computed using the well-known trapezoid rule.

(A12) $$\mathrm{Absolute}\;\mathrm{energy}\;=\sum_{i=1}^{n}x_{i}^{2},$$

(A13) $$\mathrm{Total}\;\mathrm{energy}\;=\frac{\sum_{i=1}^{n}x_{i}^{2}}{t_{n}-t_{0}},$$

(A14) $$\mathrm{Centroid}=\left\{\begin{array}{cc} \frac{T\cdot E}{\mathrm{Absolute}\mathrm{energy}}, & \mathrm{if}\;\mathrm{Absolute}\;\mathrm{energy}\neq 0\\ 0, & \mathrm{Otherwise} \end{array}\right.$$

where *n* is the size of the signal and $t_{0}$ and $t_{n}$ are initial and last time stamp of the signal (time series). In addition, (·) represents the dot product of the two vectors $T=\{t_{0},t_{1},\cdots t_{n}\}$ and $E=\{x_{0}^{2},x_{1}^{2},\cdots x_{n}^{2}\}$.

### Appendix A.11. Autocorrelation

Autocorrelation compares the similarity between a time-delayed (shifted) version of a signal to the signal itself. For periodic signals, the integer multiple delays/shifts of the signals are perfectly correlated with the signal itself [72].

### Appendix A.12. Shannon Entropy

Shannon entropy is a well-known metrics to measure the uncertainty of a random process, which can be used in non-linear and non-stationary signals [73]. In other words, Shannon entropy can be used to quantify the degree of complexity of a signal. Shannon entropy is formulated as [74]:

(A15) $$\mathrm{Shannon}\;\mathrm{entropy}\;=\sum_{i=0}^{n}\ln p\left(x_{i}\right),$$

where $p(x_{i})$ is probability of the $x_{i}$ data point such that $\sum_{i=0}^{n}p\left(x_{i}\right)=1$.

### Appendix A.13. Mean and Median Absolute Differences, Mean and Median Differences, and Sum of Absolute Differences

Mean absolute differences measures the absolute of average variability of the 1st order difference of the time series. Similarly, median absolute differences is used to calculate the median variability of the 1st order difference of the time series. These can be formulated as:

(A16) $$\mathrm{Mean}\;\mathrm{absolute}\;\mathrm{differences}\;=\frac{1}{n}\sum_{i=0}^{n}\left|x_{i+1}-x_{i}\right|,$$

(A17) $$\mathrm{Median}\;\mathrm{absolute}\;\mathrm{differences}\;=Median\left(|x_{i+1}-x_{i}|\right),\;i=1,2,\cdots ,n,$$

where *n* is the size of the time series and Median is defined in (A4). Unlike the above-mentioned two features, the mean and median differences only consider the 1st order difference of the signal without the absolute (|·|) operator. Finally, the sum of absolute differences is simply given by (A18):

(A18) $$\mathrm{Sum}\;\mathrm{of}\;\mathrm{absolute}\;\mathrm{differences}\;=\sum_{i=0}^{n}\left|x_{i+1}-x_{i}\right|,$$

where *n* is the size of the time series.

### Appendix A.14. Positive and Negative Turning Points & Zero-Crossing Rate

The positive and negative turning points count how many times a time series or a signal changes from positive to negative and from negative to positive, respectively, while zero-crossing rate measures the total number of times that the time series changes from positive to negative or vice versa.

### Appendix A.15. Peak to Peak Distance

The peak to peak distance calculates the difference between the maximum and minimum values of the time series, which is computed as:

(A19) Peak to peak distance =|Max|−|Min|,

where Max and Min are the maximum and minimum of the time series calculated as described in Appendix A.5.

### Appendix A.16. Traveled Distance

The signal travel distance is used to measure the total distance traversed by the time series (signal) using the hypotenuse between two data points, which is formulated as:

(A20) $$\mathrm{Signal}\;\mathrm{traveled}\;\mathrm{distance}\;=\sum_{i=0}^{n}\left(\sqrt{1+{\left(x_{i+1}-x_{i}\right)}^{2}}\right),$$

where *n* is the size of the time series.

### Appendix A.17. Slope of Signal

The slope of a signal is determined by fitting a linear equation to the data points (samples) [75]. Specifically, a least squares polynomial method is fitted to the data [76].

### Appendix A.18. Number of Peaks from a Subsequence

This feature looks for the biggest value among its neighbors in the subsequence. For example, a peak is to be found that is bigger than *l* neighbors to the left and *l* neighbors to the right [77].

### Appendix A.19. Mean Frequency of a Spectrogram

The mean frequency of a spectrogram is calculated by the summation of the product of spectrogram intensity and the frequency, divided by the total sum of spectrogram intensity, which is formulated as [78]:

(A21) $$\mathrm{Mean}\;\mathrm{frequency}\;\mathrm{of}\;a\;\mathrm{spectrogram}\;=\frac{\sum_{i=0}^{n_{f}}\left(S_{i}\cdot f_{i}\right)}{\sum_{i=0}^{n_{f}}S_{i}},$$

where $n_{f}$ is the number of frequency bins. Furthermore, $S_{i}$ and $f_{i}$ are the intensity and frequency at bin *i*, respectively.

### Appendix A.20. Fundamental Frequency

The fundamental frequency is often defined as the frequency that best describe the content of a signal spectrum [79].

### Appendix A.21. Human Range Energy Ratio

The human range energy ratio is calculated by finding the ratio between the energy of the signal in the frequency range of 0.6–2.5 Hz and the the whole energy of the signal.

### Appendix A.22. Linear Prediction Cepstral Coefficients and Mel-Frequency Cepstral Coefficients

The cepstrum is the computed inverse Fourier transform of the estimated signal spectrum, which can be used to find periodic structures in frequency spectra [80]. Linear prediction cepstral coefficients is computed similar to cepstrum except the smoothed auto-regressive power spectrum is used instead of estimated signal spectrum [81]. Mel-frequency cepstrum (MFC) represents the short-term power spectrum of a signal and mel-frequency cepstral coefficients are the coefficients that create an MFC [82].

### Appendix A.23. Spectral Positive Turning Points and Spectral Roll-Off and Roll-On

Spectral positive turning points counts the number of frequency amplitude changes from positive to negative values. The spectral roll-off and roll-on correspond to the frequencies where 95% and 5% of the signal magnitude can be captured below these values, respectively, [83].

### Appendix A.24. Spectral Entropy

Spectral entropy is considered as a normalised form of Shannon entropy. The power spectrum amplitude components of the time series are used for entropy evaluation [73,84]. The spectral entropy of a signal is given by:

(A22) $$\mathrm{Spectral}\;\mathrm{entropy}\;=\sum_{k=1}^{K}p_{k}\cdot \log\left(\frac{1}{p_{k}}\right),$$

where $p_{k}$ is the power of the signal (time series) at bin *k*.

### Appendix A.25. Spectral Centroid, Spread, Skewness, and Kurtosis

Spectral centroid measures the spectral center, which is defined as [85]:

(A23) $$\mu_{1}\left(t_{m}\right)=\sum_{k=1}^{K}f_{k}\cdot p_{k}\left(t_{m}\right),$$

where $f_{k}$ denotes the frequency of bin *k* and $t_{m}$ is the center of an analysis window in seconds. Spectral spread, which is also called spectral standard-deviation, calculates the spread of the spectrum around its mean value. Spectral spread can be computed as [85]:

(A24) $$\mu_{2}\left(t_{m}\right)={\left(\sum_{k=1}^{K}{\left(f_{k}-\mu_{1}\left(t_{m}\right)\right)}^{2}\cdot p_{k}\left(t_{m}\right)\right)}^{\frac{1}{2}},$$

where $f_{k}$ denotes the frequency of bin *k* and $t_{m}$ is the center of an analysis window in seconds. $\mu_{2}\left(t_{m}\right)$ is the spectral centroid at $t_{m}$ as given in (A23). Spectral skewness measures the asymmetry of the spectrum of signal (time series) around its mean value, which can be calculated as [85]:

(A25) $$\mu_{3}\left(t_{m}\right)=\frac{\left(\sum_{k=1}^{K}{\left(f_{k}-\mu_{1}\left(t_{m}\right)\right)}^{3}\cdot p_{k}\left(t_{m}\right)\right)}{\mu_{2}{\left(t_{m}\right)}^{3}},$$

where $f_{k}$ denotes the frequency of bin *k* and $t_{m}$ is the center of an analysis window in seconds. $\mu_{1}\left(t_{m}\right)$ and $\mu_{2}\left(t_{m}\right)$ are the spectral centroid and spread at $t_{m}$ as given in (A23) and (A24). $\mu_{3}$=0 corresponds to a symmetric distribution, while $\mu_{3}>$ 0 and $\mu_{3}<$ 0 indicate more energy at lower and higher frequencies with respect to the mean value, respectively. Spectral kurtosis measures the flatness of the spectrum of signal (time series) around its mean value, which can be formulated as [85]:

(A26) $$\mu_{4}\left(t_{m}\right)=\frac{\left(\sum_{k=1}^{K}{\left(f_{k}-\mu_{1}\left(t_{m}\right)\right)}^{4}\cdot p_{k}\left(t_{m}\right)\right)}{\mu_{2}{\left(t_{m}\right)}^{4}},$$

where $f_{k}$ denotes the frequency of bin *k* and $t_{m}$ is the center of an analysis window in seconds. $\mu_{1}\left(t_{m}\right)$ and $\mu_{2}\left(t_{m}\right)$ are the spectral centroid and spread at $t_{m}$ as given in (A23) and (A24). $\mu_{4}$ = 0 corresponds to a normal distribution, while $\mu_{4}>$ 0 and $\mu_{4}<$ 0 indicate a wider and narrower distribution, respectively.

### Appendix A.26. Spectral Slope, Decrease, Variation, and Distance

Spectral slope is calculated applying a linear regression over the spectral amplitude values. There is a linear correlation between spectral slope and spectral centroid. Spectral decrease was proposed by [86] and it averages the set of spectral slopes between frequency $f_{k}$ and $f_{1}$ [86]. Spectral variation quantifies the spectral shape changes over time, which is defined as one minus the normalized correlation between the successive time frames [86]. Spectral distance calculates the cumulative sum of distances at different frequencies ($f_{k}$) with respect to the linear regression.

### Appendix A.27. Wavelet Entropy and Energy

Wavelet entropy calculates shannon entropy of continuous wavelet transform (CWT) [87]. Suppose that a set of wavelet coefficients is given as $W(a_{i},t),i=1,2,\cdots ,M$. The wavelet entropy is computed as [88]:

(A27) $$\begin{array}{c} \mathrm{Wavelet}\mathrm{entropy}=-\sum_{i=1}^{M}d_{i}\cdot \log d_{i},\\ s.t.\;d_{i}=\frac{\left|W(a_{i},t)\right|}{\sum_{j=1}^{M}W\left(a_{j},t\right)}. \end{array}$$

where *a* is the scale parameter of wavelet coefficients. Furthermore, wavelet energy is simply the sum of the squares of the absolute values of wavelet coefficients $W(a_{i},t)$.

### Appendix A.28. Wavelet Absolute Mean, Standard Deviation, and Variance

Wavelet absolute mean computes the absolute mean value of wavelet coefficients. Wavelet standard deviation and variance calculates the standard deviation and variance of wavelet coefficients, respectively.

### Appendix A.29. Maximum Power Spectrum Density and Power Spectrum Density Bandwidth

Welch’s method [89] is used to estimate the power spectral density of the the signal (time series). This method estimate the power spectral density by dividing the time series into overlapping segments. Afterwards, it computes a periodogram corresponds to each segment, which are then averaged. However, power spectrum density bandwidth represents the frequency band width, which contains 95% of the signal power.

### Appendix A.30. Maximum and Median Frequency

Maximum frequency is considered as 95% of the cumulative sum of different frequency bands resulting from the Fourier transform of the signal. Median frequency is considered the frequency in which the signal power spectrum is split into two equal regions in terms of amplitude [90]. In other words, median frequency is equal to half of the total power of the signal.

## References

[1] WHO for Europe. Strategy and Action Plan for Healthy Ageing in Europe, 2012–2020.

[2] Chodzko-Zajko W.J., Proctor D.N., Singh M.A.F., Minson C.T., Nigg C.R., Salem G.J., Skinner J.S. (2009). Exercise and physical activity for older adults. Med. Sci. Sport. Exerc..

[3] WHO, Geneva, Switzerland. Global Recommendations on Physical Activity for Health.

[4] Allen F.R., Ambikairajah E., Lovell N.H., Celler B.G. (2006). Classification of a known sequence of motions and postures from accelerometry data using adapted Gaussian mixture models. Physiol. Meas..

[5] Sekine M., Tamura T., Akay M., Fujimoto T., Togawa T., Fukui Y. (2002). Discrimination of walking patterns using wavelet-based fractal analysis. IEEE Trans. Neural Syst. Rehabil..

[6] Kamišalić A., Fister Jr I., Turkanović M., Karakatič S. (2018). Sensors and functionalities of non-invasive wrist-wearable devices: A review. Sensors.

[7] Cleland I., Kikhia B., Nugent C., Boytsov A., Hallberg J., Synnes K., McClean S., Finlay D. (2013). Optimal placement of accelerometers for the detection of everyday activities. Sensors.

[8] Khan A.M., Lee Y.K., Lee S.Y., Kim T.S. (2010). A triaxial accelerometer-based physical-activity recognition via augmented-signal features and a hierarchical recognizer. IEEE Trans. Inf. Technol. Biomed..

[9] Leutheuser H., Schuldhaus D., Eskofier B.M. (2013). Hierarchical, multi-sensor based classification of daily life activities: Comparison with state-of-the-art algorithms using a benchmark dataset. PLoS ONE.

[10] Arif M., Bilal M., Kattan A., Ahamed S.I. (2014). Better physical activity classification using smartphone acceleration sensor. J. Med Syst..

[11] Shoaib M., Bosch S., Incel O.D., Scholten H., Havinga P.J. (2016). Complex human activity recognition using smartphone and wrist-worn motion sensors. Sensors.

[12] Del Rosario M.B., Wang K., Wang J., Liu Y., Brodie M., Delbaere K., Lovell N.H., Lord S.R., Redmond S.J. (2014). A comparison of activity classification in younger and older cohorts using a smartphone. Physiol. Meas..

[13] Usmani S., Saboor A., Haris M., Khan M.A., Park H. (2021). Latest research trends in fall detection and prevention using machine learning: A systematic review. Sensors.

[14] Preece S.J., Goulermas J.Y., Kenney L.P., Howard D. (2008). A comparison of feature extraction methods for the classification of dynamic activities from accelerometer data. IEEE Trans. Biomed. Eng..

[15] Bao L., Intille S.S. Activity recognition from user-annotated acceleration data. Proceedings of the International Conference on Pervasive Computing.

[16] Guiry J.J., van de Ven P., Nelson J., Warmerdam L., Riper H. (2014). Activity recognition with smartphone support. Med Eng. Phys..

[17] Trabelsi D., Mohammed S., Chamroukhi F., Oukhellou L., Amirat Y. (2013). An unsupervised approach for automatic activity recognition based on hidden Markov model regression. IEEE Trans. Autom. Sci. Eng..

[18] Ganea R., Paraschiv-lonescu A., Aminian K. (2012). Detection and classification of postural transitions in real-world conditions. IEEE Trans. Neural Syst. Rehabil..

[19] Pedrero-Sánchez J.F., Belda-Lois J.M., Serra-Añó P., Inglés M., López-Pascual J. (2022). Classification of healthy, Alzheimer and Parkinson populations with a multi-branch neural network. Biomed. Signal Process. Control.

[20] Najafi B., Aminian K., Paraschiv-Ionescu A., Loew F., Bula C.J., Robert P. (2003). Ambulatory system for human motion analysis using a kinematic sensor: Monitoring of daily physical activity in the elderly. IEEE Trans. Biomed. Eng..

[21] Rehman R.Z.U., Del Din S., Guan Y., Yarnall A.J., Shi J.Q., Rochester L. (2019). Selecting clinically relevant gait characteristics for classification of early parkinson’s disease: A comprehensive machine learning approach. Sci. Rep..

[22] Kwon S.B., Ku Y., Han H.S., Lee M.C., Kim H.C., Ro D.H. (2020). A machine learning-based diagnostic model associated with knee osteoarthritis severity. Sci. Rep..

[23] Awais M., Mellone S., Chiari L. Physical activity classification meets daily life: Review on existing methodologies and open challenges. Proceedings of the 2015 37th Annual International Conference of the IEEE Engineering in Medicine and Biology Society (EMBC).

[24] Awais M., Chiari L., Ihlen E.A., Helbostad J.L., Palmerini L. (2021). Classical Machine Learning Versus Deep Learning for the Older Adults Free-Living Activity Classification. Sensors.

[25] Scherder E., Eggermont L., Swaab D., van Heuvelen M., Kamsma Y., de Greef M., van Wijck R., Mulder T. (2007). Gait in ageing and associated dementias; its relationship with cognition. Neurosci. Biobehav. Rev..

[26] Della Sala S., Spinnler H., Venneri A. (2004). Walking difficulties in patients with Alzheimer’s disease might originate from gait apraxia. J. Neurol. Neurosurg. Psychiatry.

[27] Polikar R. (2006). Ensemble based systems in decision making. IEEE Circuits Syst. Mag..

[28] Okey O.D., Maidin S.S., Adasme P., Lopes Rosa R., Saadi M., Carrillo Melgarejo D., Zegarra Rodríguez D. (2022). BoostedEnML: Efficient Technique for Detecting Cyberattacks in IoT Systems Using Boosted Ensemble Machine Learning. Sensors.

[29] Wang X., Zhang L., Zhao K., Ding X., Yu M. (2022). MFDroid: A Stacking Ensemble Learning Framework for Android Malware Detection. Sensors.

[30] Dutta V., Choraś M., Pawlicki M., Kozik R. (2020). A deep learning ensemble for network anomaly and cyber-attack detection. Sensors.

[31] Alsaedi M., Ghaleb F.A., Saeed F., Ahmad J., Alasli M. (2022). Cyber Threat Intelligence-Based Malicious URL Detection Model Using Ensemble Learning. Sensors.

[32] Talaei Khoei T., Ismail S., Kaabouch N. (2022). Dynamic selection techniques for detecting GPS spoofing attacks on UAVs. Sensors.

[33] Derhab A., Guerroumi M., Gumaei A., Maglaras L., Ferrag M.A., Mukherjee M., Khan F.A. (2019). Blockchain and random subspace learning-based IDS for SDN-enabled industrial IoT security. Sensors.

[34] Yuan J., Liu L., Yang Z., Zhang Y. (2020). Tool wear condition monitoring by combining variational mode decomposition and ensemble learning. Sensors.

[35] Xu G., Liu M., Jiang Z., Söffker D., Shen W. (2019). Bearing fault diagnosis method based on deep convolutional neural network and random forest ensemble learning. Sensors.

[36] Beretta M., Julian A., Sepulveda J., Cusidó J., Porro O. (2021). An ensemble learning solution for predictive maintenance of wind turbines main bearing. Sensors.

[37] Ai S., Chakravorty A., Rong C. (2019). Household power demand prediction using evolutionary ensemble neural network pool with multiple network structures. Sensors.

[38] Ku Abd. Rahim K.N., Elamvazuthi I., Izhar L.I., Capi G. (2018). Classification of human daily activities using ensemble methods based on smartphone inertial sensors. Sensors.

[39] Mahendran N., Vincent D.R., Srinivasan K., Chang C.Y., Garg A., Gao L., Reina D.G. (2019). Sensor-assisted weighted average ensemble model for detecting major depressive disorder. Sensors.

[40] Aljihmani L., Kerdjidj O., Zhu Y., Mehta R.K., Erraguntla M., Sasangohar F., Qaraqe K. (2020). Classification of Fatigue Phases in Healthy and Diabetic Adults Using Wearable Sensor. Sensors.

[41] Wall C., Zhang L., Yu Y., Kumar A., Gao R. (2022). A deep ensemble neural network with attention mechanisms for lung abnormality classification using audio inputs. Sensors.

[42] Peimankar A., Puthusserypady S. An ensemble of deep recurrent neural networks for p-wave detection in electrocardiogram. Proceedings of the ICASSP 2019-2019 IEEE International Conference on Acoustics, Speech and Signal Processing (ICASSP).

[43] Peimankar A., Puthusserypady S. Ensemble learning for detection of short episodes of atrial fibrillation. Proceedings of the 2018 26th European Signal Processing Conference (EUSIPCO).

[44] Resmini R., Silva L., Araujo A.S., Medeiros P., Muchaluat-Saade D., Conci A. (2021). Combining genetic algorithms and SVM for breast cancer diagnosis using infrared thermography. Sensors.

[45] Dissanayake T., Rajapaksha Y., Ragel R., Nawinne I. (2019). An ensemble learning approach for electrocardiogram sensor based human emotion recognition. Sensors.

[46] Luo J., Gao X., Zhu X., Wang B., Lu N., Wang J. (2020). Motor imagery EEG classification based on ensemble support vector learning. Comput. Methods Programs Biomed..

[47] Huang J.C., Tsai Y.C., Wu P.Y., Lien Y.H., Chien C.Y., Kuo C.F., Hung J.F., Chen S.C., Kuo C.H. (2020). Predictive modeling of blood pressure during hemodialysis: A comparison of linear model, random forest, support vector regression, XGBoost, LASSO regression and ensemble method. Comput. Methods Programs Biomed..

[48] Kennedy J., Eberhart R. Particle swarm optimization. Proceedings of the ICNN’95-International Conference on Neural Networks.

[49] SENS Innovation ApS SENS Innovation ApS. https://sens.dk/en/.

[50] Barandas M., Folgado D., Fernandes L., Santos S., Abreu M., Bota P., Liu H., Schultz T., Gamboa H. (2020). TSFEL: Time series feature extraction library. SoftwareX.

[51] Kira K., Rendell L.A. (1992). A practical approach to feature selection. Machine Learning Proceedings 1992.

[52] Guyon I., Elisseeff A. (2003). An introduction to variable and feature selection. J. Mach. Learn. Res..

[53] Peimankar A., Weddell S.J., Jalal T., Lapthorn A.C. (2017). Evolutionary multi-objective fault diagnosis of power transformers. Swarm Evol. Comput..

[54] Peimankar A., Weddell S.J., Jalal T., Lapthorn A.C. (2018). Multi-objective ensemble forecasting with an application to power transformers. Appl. Soft Comput..

[55] Eberhart R.C., Shi Y., Kennedy J. (2001). Swarm Intelligence.

[56] Shi Y. Particle swarm optimization: Developments, applications and resources. Proceedings of the 2001 Congress on Evolutionary Computation (IEEE Cat. No. 01TH8546).

[57] Fix E., Hodges J.L. (1989). Discriminatory analysis. Nonparametric discrimination: Consistency properties. Int. Stat. Rev. Int. De Statistique.

[58] Altman N.S. (1992). An introduction to kernel and nearest-neighbor nonparametric regression. Am. Stat..

[59] Breiman L., Friedman J.H., Olshen R.A., Stone C.J. (2017). Classification and Regression Trees.

[60] Ho T.K. Random decision forests. Proceedings of the 3rd International Conference on Document Analysis and Recognition.

[61] Breiman L. (2001). Random forests. Mach. Learn..

[62] Chen T., Guestrin C. Xgboost: A scalable tree boosting system. Proceedings of the 22nd ACM Sigkdd International Conference on Knowledge Discovery and Data Mining.

[63] Friedman J.H. (2002). Stochastic gradient boosting. Comput. Stat. Data Anal..

[64] Wolpert D.H. (1992). Stacked generalization. Neural Netw..

[65] Ting K.M., Witten I.H. Stacked generalization: When does it work?. Proceedings of the Fifteenth International Joint Conference on Artifical Intelligence.

[66] Ting K.M., Witten I.H. (1999). Issues in stacked generalization. J. Artif. Intell. Res..

[67] He H., Bai Y., Garcia E.A., Li S. ADASYN: Adaptive synthetic sampling approach for imbalanced learning. Proceedings of the 2008 IEEE International Joint Conference on Neural Networks (IEEE World Congress on Computational Intelligence).

[68] Pedregosa F., Varoquaux G., Gramfort A., Michel V., Thirion B., Grisel O., Blondel M., Prettenhofer P., Weiss R., Dubourg V. (2011). Scikit-learn: Machine learning in Python. J. Mach. Learn. Res..

[69] Harris C.R., Millman K.J., Van Der Walt S.J., Gommers R., Virtanen P., Cournapeau D., Wieser E., Taylor J., Berg S., Smith N.J. (2020). Array programming with NumPy. Nature.

[70] McKinney W. Data structures for statistical computing in python. Proceedings of the 9th Python in Science Conference.

[71] Hunter J.D. (2007). Matplotlib: A 2D graphics environment. Comput. Sci. Eng..

[72] Gubner J.A. (2006). Probability and Random Processes for Electrical and Computer Engineers.

[73] Shannon C.E. (1948). A mathematical theory of communication. Bell Syst. Tech. J..

[74] Pathria R.K., Beale P.D. (2011). Statistical Mechanics.

[75] Sandra L. (1994). PHB Practical Handbook of Curve Fitting.

[76] Humpherys J., Jarvis T.J., Evans E.J. (2017). Foundations of Applied Mathematics, Volume I: Mathematical Analysis.

[77] Christ M., Braun N., Neuffer J., Kempa-Liehr A.W. (2018). Time series feature extraction on basis of scalable hypothesis tests (tsfresh–a python package). Neurocomputing.

[78] Cohen L. (1995). Time-Frequency Analysis.

[79] Kwong S., Gang W., Zheng O.Y.J. Fundamental frequency estimation based on adaptive time-averaging Wigner-Ville distribution. Proceedings of the IEEE-SP International Symposium on Time-Frequency and Time-Scale Analysis.

[80] Bogert B.P. (1963). The quefrency alanysis of time series for echoes; Cepstrum, pseudo-autocovariance, cross-cepstrum and saphe cracking. Time Ser. Anal..

[81] Reynolds D.A. (1995). Speaker identification and verification using Gaussian mixture speaker models. Speech Commun..

[82] Xu M., Duan L.Y., Cai J., Chia L.T., Xu C., Tian Q. HMM-based audio keyword generation. Proceedings of the Pacific-Rim Conference on Multimedia.

[83] Peeters G. (2004). A large set of audio features for sound description (similarity and classification) in the CUIDADO project. CUIDADO Ist Proj. Rep..

[84] Fell J., Röschke J., Mann K., Schäffner C. (1996). Discrimination of sleep stages: A comparison between spectral and nonlinear EEG measures. Electroencephalogr. Clin. Neurophysiol..

[85] Peeters G., Giordano B.L., Susini P., Misdariis N., McAdams S. (2011). The timbre toolbox: Extracting audio descriptors from musical signals. J. Acoust. Soc. Am..

[86] Krimphoff J., McAdams S., Winsberg S., Petit H., Bakchine S., Dubois B., Laurent B., Montagne B., Touchon J., Robert P. (1994). Characterization of the timbre of complex sounds. 2. Acoustic analysis and psychophysical quantification. J. Phys..

[87] Addison P.S. (2017). The Illustrated Wavelet Transform Handbook: Introductory Theory and Applications in Science, Engineering, Medicine and Finance.

[88] Yan B., Miyamoto A., Brühwiler E. (2006). Wavelet transform-based modal parameter identification considering uncertainty. J. Sound Vib..

[89] Welch P. (1967). The use of fast Fourier transform for the estimation of power spectra: A method based on time averaging over short, modified periodograms. IEEE Trans. Audio Electroacoust..

[90] Phinyomark A., Phukpattaranont P., Limsakul C. (2012). Feature reduction and selection for EMG signal classification. Expert Syst. Appl..

